# Longitudinal study on oral shedding of human betaherpesviruses 6 and 7 in renal transplant recipients reveals active replication

**DOI:** 10.1080/20002297.2020.1785801

**Published:** 2020-06-30

**Authors:** Jéssica Vasques Raposo, Dmitry José De Santana Sarmento, Rafaela Barbosa Da Silva Pinto, Amanda Oliveira Lopes, Marina Gallottini, Tânia Regina Tozetto-Mendoza, Paulo Henrique Braz-Silva, Vanessa Salete de Paula

**Affiliations:** a Instituto Oswaldo Cruz – IOC/FIOCRUZ – Fundação Oswaldo Cruz, Rio De Janeiro, Brasil; b Departamento de Estomatologia, Faculdade de Odontologia da Universidade de São Paulo – FOUSP, São Paulo, SP, Brasil; c Laboratório de Virologia, Instituto de Medicina Tropical de São Paulo – IMTSP – Faculdade de Medicina da Universidade de São Paulo, São Paulo, SP, Brasil

**Keywords:** Herpesviruses, qPCR, roseolovirus, saliva, renal transplantation

## Abstract

**Backgroung:**

Roseolovirus latency and persistence in salivary glands that are frequently reactivated after renal transplantation to cause infection have been reported. However, limited information is available on the persistence and excretion of HHV-6 and HHV-7 during and after transplant.

**Methods:**

32 renal transplant recipients were followed up before (T1) and after transplant (T2 and T3) and viral replication (via assessment of mRNA) in oral fluid samples investigated. Roseolovirus DNA was detected and quantified via multiplex qPCR. For evaluation of mRNA replication, positive samples were subjected to nested RT-PCR.

**Results:**

Viral replication of HHV-7 was significantly increased during T3 (72.9%), compared to the pre-transplant period T1 (25%; McNemar Test, p= 0.001). Analysis of the viral replicative to quantitative ratio disclosed ahigher number of DNA copies (>10^6^) in positive cases of replication (p < 0.001). Astrong positive correlation (Spearman correlation coefficient = 0.781; p< 0.001) was evident between viral quantities of Roseoloviruses.

**Conclusion:**

Our findings consistently suggest that the salivary gland is an important site of active and persistent infection by roseoloviruses. In view of the increasing problem of Roseoloviruses, pre- and post-transplantation, viral surveillance and monitoring of active replication are pivotal steps for effective screening and treatment of renal transplant patients.

## Introduction

Human herpesvirus 6 (HHV-6A/B) and human herpesvirus 7 (HHV-7) belong to the genus *Roseolovirus* and subfamily *Betaherpesvirinae*. Although CD4 + T lymphocytes have been identified as the preferred replication site, HHV-6 is characterized by widespread tropism in multiple human cell types and classified into two closely related variant groups, HHV-6A and HHV-6B, which are currently recognized as distinct herpesvirus species. HHV-6B is reported as the major causative agent of sudden rash while no clear association with HHV-6A has been established [1].

HHV-7 was isolated in 1990 from a healthy individual whose cells were stimulated with CD3 antibody and incubated with interleukin-2 (IL-2) [1]. HHV-7 is one of the causative agents of sudden rash [2] and associated with febrile seizures in young children [3]. HHV-6A/B and HHV-7 are ubiquitous, and >85% of adults develop antibodies against both viruses [4]. HHV-6A, HHV-6B and HHV-7 infections correspond mainly to reactivation of latent viruses during a period of immunodeficiency. The associated symptoms and diseases include rash, cytopenia, pneumonitis, hepatitis and encephalitis in organ recipients as well as colitis and retinitis in HIV-infected patients [5,6]. Roseoloviruses have additionally been detected in patients with chronic kidney disease after transplantation [4]. Viral reactivations often trigger serious diseases in solid organ recipients, such as kidney transplant patients, with negative effects on outcomes [4,7].

Patient survival after renal transplantation has been frequently investigated over the last few decades [8]. Infection is reported as the most common non-cardiovascular cause of mortality following kidney transplantation, accounting for ~15% to 20% deaths [9]. In both pediatric and adult renal transplants, HHV-6 and HHV-7 are often reactivated. Immunosuppression and various transplant-associated treatments are also reported to promote the reactivation of HHV-6 and HHV-7 [10]. Viral reactivation in immunocompromised patients is commonly associated with fever, rash, encephalitis, and bone marrow suppression [11].

Limited information is currently available on the persistence and excretion of viruses during and after infection. Salivary glands have been identified as the potential site of roseolovirus latency and persistence [12]. Studies to date suggest that both HHV-6 and HHV-7 viruses persist in the host and are eliminated in saliva throughout life [13].

Lack of knowledge on the prevalence, reactivation and latency sites of viruses hinder treatment, as underreported cases in solid organ recipients often results in serious illness or even death. Considering the increasing problem of pre- and post-renal transplant HHV-6 and HHV-7 infections, monitoring of the detection (DNA) and active replication (mRNA) phases is critical. Data obtained on HHV-6 and HHV-7 to date do not distinguish between viral shedding and active replication [14,15]. In this context, we have aimed to quantify roseolovirus levels in oral fluid samples and detect intermediate replicative forms from transplanted patients in the current study.

## Materials and methods

### Study population

This study was approved by the Ethics committee of the Kidney and Hypertension Hospital (Osvaldo Ramos Foundation approval protocol no. 2.362.239). Our cohort study included patients over 18 years of age from a referral renal transplantation service who underwent single kidney transplantation in Brazil. Exclusion criteria included another type of organ transplant, kidney transplant associated with that of another organ, immunosuppressive therapy before initiation of the study, HIV positivity or a neurological disease that could lead to complications in data interpretation. Patients who dropped out or had inconclusive viral detection results in salivary samples were also excluded.

All patients received immunosuppressive therapy and six patients used ganciclovir for treatment of CMV disease, consisted of intravenous 5 mg/kg b.i.d. ganciclovir, adjusted for renal function [16].

The periodontal condition was evaluated by Community Periodontal Index of Treatment Needs (CPITN). Scoring and treatment criteria were defined as follows: 0 = healthy periodontium, 1 = gingival bleeding during probing, 2 = dental calculus, 3 = periodontal pockets (with interproximal attachment loss) 3–5 mm, 4 = periodontal pockets of 6 mm depth [17]. The mean score value was used for statistical analysis.

To determine the frequency and active replication of HHV-6 and HHV-7 in renal transplant patients, a longitudinal study was conducted on 32 patients enrolled without preliminary screening who underwent consecutive transplantation between January 2017 and January 2018. For oral fluid collection, patients were instructed to rinse with 5 mL of commercial mouthwash (Listerine®, Johnson & Johnson, Sao Paulo, Brazil) for 30 seconds and deposit into a 50 mL Falcon collector tube. After collection and identification, oral samples were centrifuged at 800 rpm in a conical tube. Aliquots (200 μL) of each sample were placed in cryotubes and stored at −80°C until laboratory analysis. Collections were performed at three time-points: T1 (up to 24 hours before transplant), T2 (15 to 20 days after transplant) and T3 (45 to 60 days after transplant).

### Extraction of nucleic acids

Roseolovirus DNA and RNA were extracted using the High Pure Viral Nucleic Acid kit (Roche Diagnostics, Mannheim, Germany) according to the manufacturer’s protocols. This technique involves viral lysis by incubating samples with binding buffer together with proteinase K enzyme. Subsequently, nucleic acids specifically bound the fiberglass in the column matrix in the presence of chaotropic salts. After washing out contaminants, nucleic acid was eluted with a low-salt solution. DNA and RNA samples were stored at −70ºC until processing.

### Real-time multiplex qPCR analysis

The quantitative real-time PCR assay was applied to measure viral loads [18] using U56 and U37 regions to target HHV-6 A/B and HHV-7, respectively. Primers and oligonucleotide TaqMan probes are presented in Table 1. Multiplex qPCR was performed in a reaction mixture comprising 1 µL 25xPCR Enzyme Mix (Life Technologies, California, USA), 2.5 µL of each oligonucleotide (3 µM), 2 µL probe (0.4 µM), 12.5 µL of 1x PCR Buffer (Life Technologies, California, USA) and 5 µL DNA. A synthetic standard curve was used for absolute quantification of viral DNA [18].

**Table 1.** Sequences of primers and DNA probes used for standard curves for qPCR.

		HHV-6 sequence (5´-3´)	HHV-7 sequence (5´-3´)
Probes	**VIC-**TTAGATGGTGGTGAGCTGGGATCGGT- NFQ-MGB		**NED –** CTCGCAGATTGCTTGTTGGCCATG- NFQ-MGB
Primers (Sense)	AAAGACCTAAATTGCCGCTACCT		CGGAAGTCACTGGAGTAATGACAA
Primers (Anti-sense)	GCAAGCTCATGAACATCGTCA		CCAATCCTTCCGAAACCGAT
Standard Curve probe	TTCGTGCAAGCTCATGAACATCGTCACGTATACCGATCCCAGCTCACCACCATCTAAATGCGTAGGTAGCGGCAATTTAGGTCTTTCTGATA		TTCGTCCAATCCTTCCGAAACCGATCGTATCATGGCCAACAAGCAATCTGCGAGATGCGTTTGTCATTACTCCAGTGACTTCCGCTGATA

### 2.4. mRNA detection via nested RT-PCR

To evaluate viral replication, HHV-6 and HHV-7 U79/80 regions were detected using nested RT-PCR. U79/80 were selected as the template sequences owing to their important role in viral DNA replication [19].

Initially, genomic DNA was removed from RNA preparations by adding 1 μg RNA, 10X reaction buffer with MgCl_2,_ and 1 U DNase I (Promega; Madison, WI, USA), according to the manufacturer’s instructions. For detection of mRNA, cDNA was synthesized by adding 10 mM sense primer, 500 U EZ-rTthRNA, 10 μL of 5X EZ Buffer, 10 mM DNTP, 2 μM MgCl_2_ and 21 μL H_2_O (Applied Biosystems, Foster City, CA, USA) to the mixture and incubating at 60°C for 1 h. The reaction was inactivated at 95°C for 10 minutes.

Nested RT-PCR was performed in a reaction mixture comprising 2 U *Taq* Platinum enzyme, 10 mM DNTP, 10x PCR buffer and 50 mM MgCl_2_, HHV-6, and the appropriate primer pairs (0.2 mM) (Table 2). The reaction was conducted for 35 cycles (denaturation at 95°C for 30 s, annealing at 62°C (first round) and 53°C (second round) for 30 s, and elongation at 75°C for 1 min). For HHV-7, the reaction conducted with 0.2 mM primer pairs for 35 cycles (denaturation at 95°C for 30 s, annealing at 56°C (first round) and 55°C (second round) for 30 s, and elongation at 75°C for 1 min). A 10 min elongation step was performed after the final cycle to ensure complete polymerization.

**Table 2.** Sequences of nPCR primers.

	**HHV-6 sequence (5´- 3´)**	**HHV-7 sequence (5´- 3´)**
Primers – (Sense) F1	5ʹ-CTCGGAGCCCGAGCTACGAAATCTG-3’	5ʹ-TTGCGGAGGATAGAGAATATGGAAC-3’
Primers – (Anti- Sense) R1	5ʹ-GCAACAAGTCTGGCCCTAAGCTG-3’	5ʹ-TCGTTCTGCATGTTCTTTTTCAAAC-3’
Primers (sense) F2	5ʹ-CGATGAACACAGAAAACGCTCG-3’	5ʹ-AGAAACTCTTCTGGGAGCAGTTCA-3’
Primers (Anti-sense) R2	5ʹ-GTAGAAGTCGGTGCTATGCCATC-3’	5ʹ-CGACACCAGAGCTGTGGCAA-3’

### Statistical analysis

Results were analyzed using SPSS software version 17.0 (SPSS, Inc., Chicago, IL, USA). Viral replication between collection periods was analyzed using the McNemar test. The Mann–Whitney test was applied to associate viral replication and ganciclovir usage with HHV-6 and HHV-7 and with CPITN and Kappa test used to evaluate the correlation between HHV-6 and the HHV-7 replication. Spearman correlation was applied for determining the correlation between HHV-6 and HHV-7 viral quantities and Fisher’s exact test to analyze associations of viral replication of herpesviruses with the use of ganciclovir. The adopted significance level was 5%.

## Results

### Demographic characteristics of patients

The clinical and epidemiological profiles of patients were white (43.8%), mean age of 42.00 ± 11.48 years, and no evident gender bias. Age, dialysis time, gender, skin color, donor type, immunosuppressive regimen, and periodontal condition (CPITN – Community Periodontal Index of Treatment Needs) (p > 0.005) were not significantly associated with viral replication or quantification at all the time-points evaluated.

### Real-time multiplex PCR for detection and quantification of roseoloviruses

**Figure 1.**
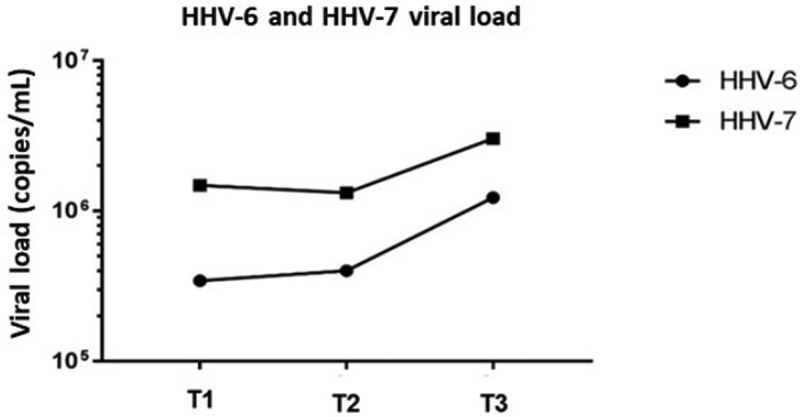
HHV-6 and HHV-7 viral loads at pre and post-transplantation periods in oral fluids of renal transplant patients (T1: Pre transplantation and without immunosuppression treatment; T2: 15–20 days after transplantation; T3: 45 to 60 days post-transplantation).

Viral load was high during T1, T2 and T3 time periods, with mean values of 6.51E+05 copies/mL and 1.93E+06 copies/mL for HHV-6 and HHV-7, respectively (Figure 1). Furthermore, 28 (87.5%) samples were positive for HHV-6 at T1, 28 (87.5%) at T2 and 27 (84.3%) at T3. In total, 30 (94%) samples were positive for HHV-7 at T1, 32 (100%) at T2 and 30 (94%) at T3. Viral DNA was simultaneously detected in 26 samples (81%) at T1, 28 samples (87%) at T2 and 27 samples (84%) at T3.

### mRNA detection via nested RT-PCR

**Figure 2.**
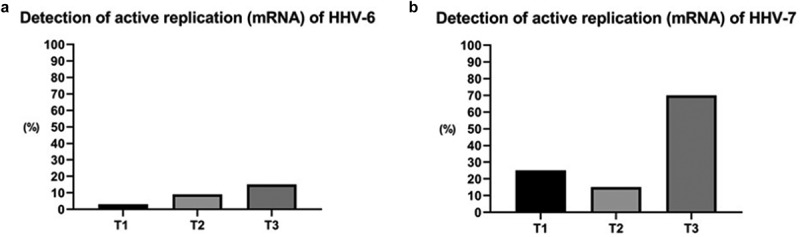
Detection of actively replicating HHV-6 and HHV-7 based on mRNA analysis in oral fluid before and after kidney transplantation. (T1: pre-transplant and without immunosuppressive treatment; T2: 15–20 days after transplantation; T3: 45 to 60 days after transplantation).

Over the three time periods examined, replication of HHV-6 and HHV-7 was increased 45 days after transplantation. HHV-6 mRNA was detected in one (3%) sample at T1, three (9%) samples at T2, and five (15%) samples at T3. HHV-7 mRNA was detected in eight (25%), five (15%) and 24 (70%) samples, respectively, at the three selected time periods (Figure 2).

### Statistical analysis

Viral replication and quantification were not statistically associated with age, dialysis time, gender, skin color, donor type, immunosuppressive regimen or periodontal condition (CPITN) (p > 0.005) at the selected time periods of transplantation.

HHV-7 displayed a significant increase in replication during the 45–60 days post-transplant period (71.9%), compared to the pre-transplant period (25%) (McNemar Test, p = 0.001). HHV-6 additionally presented increased replication during the evaluated periods, but not to a significant extent (p > 0.05) (Table 3).

**Table 3.** Association of HHV-6 and HHV-7 replication with transplant times.

**Virus**	**Time period**	**Positive** **n (%)**	**Negative** **n (%)**	**TOTAL**	**p^[1]^**
HHV-6	Pre-transplantation	1 (3.1)	31 (96.9)	32 (100)	
	15–20 days after transplantation	3 (9.4)	29 (90.6)	32 (100)	0.625
	45 to 60 days after transplantation	6 (18.8)	26 (81.3)	32 (100)	0.063
HHV-7	Pre-transplantation	8 [25]	24 (75)	32 (100)	
	15–20 days after transplantation	5 (15.6)	27 (84.4)	32 (100)	0.508
	45 to 60 days after transplantation	23 (71.9)	9 (28.1)	32 (100)	0.001*

[1] McNemar Test, ‘p’ values correspond to the significance of pre-transplant data relative to 15–20 days and 45–60 days after transplantation.

* Statistical significance (p < 0.05).

Table 4 presents the relationship between HHV-6 and HHV-7 DNA quantities and the detection of mRNA replicative intermediates. In samples where viral loads were >10^6^ copies/mL DNA, mRNA was detectable (p < 0.001).

**Table 4.** Relationship between HHV-6 and HHV-7 DNA levels and mRNA detection.

**mRNA Herpesvirus**		**Quantitation**			***p*^(b)^**
		**Mean** ± **Standard deviation** **(DNA copies/mL)**	**N^a^**	**Rank Mean**	
HHV-6	Negative	4.58 10^5^ ± 1.22 10^6^	86	46.64	0.055
	Positive	1.45 10^6^ ± 2.80 10^6^	10	64.50	
HHV-7	Negative	7.48 10^5^ ± 1.24 10^6^	60	40.38	<0.001*
	Positive	3.91 106 ± 7.63 10^6^	36	62.04	
					

^a^represents the number of samples evaluated. (b) Mann-Whitney Test * Statistical significance (p < 0.05).

**Figure 3.**
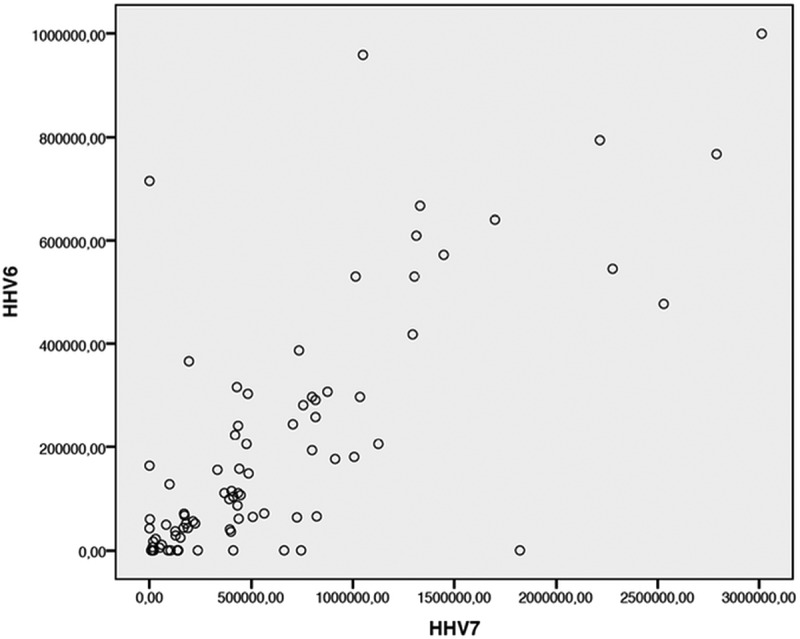
Correlations between HHV-6 and HHV-7 viral DNA quantities (Spearman correlation: N = 96, Correlation coefficient = 0.781, p < 0.001).

Comparison of viral loads revealed a strong positive correlation (Spearman correlation coefficient = 0.781; p < 0.001) between the viral DNA quantities of HHV-6 and HHV-7 (Figure 3).

Most of the patients (46.9%) used Tacrolimus, Azathioprine and Prednisone as immunosuppressive drugs (Table 5). The type of treatment was not associated with viral replication of HHV-6 or HHV-7 (Fisher’s Exact Test, p = 0.05). The use of ganciclovir induced a decrease in HHV-6 (Mann–Whitney Test, p = 0.431) and HHV-7 quantities (Mann–Whitney Test, p = 0.209), but not to a significant extent (data not shown).

**Table 5.** Immunosuppressive regimens adopted for the study participants.

Immunosuppressive regimen	n	%
Tacrolimus + Azathioprine + Prednisone	15	46.9
Cyclosporine + Azathioprine + Prednisone	5	15.6
Tacrolimus + Everolimus + Prednisone	7	21.9
Tacrolimus + Mycophenolate sodium + Prednisone	5	15.6
TOTAL	32	100

Note: No regimen or individual use of medication was associated with viral replication of HHV-6 or HHV-7 (Fisher’s Exact Test, p > 0.05).

## Discussion

Roseolovirus co-infection may be considered an evolutionary factor underlying serious diseases [20]. Recently, both HHV-6 and HHV-7 were detected in transplant patients who died of acute liver failure with no defined etiology [7]. Here, we observed high prevalence rates of HHV-6 and HHV-7 before and after transplantation as well as viral co-infection. Persistent DNA in oral fluid represents the reactivation of latent viruses in both immunocompetent and immunosuppressed patients [14]. The prevalence of HHV-6 and HHV-7 in oral fluid samples has been described previously, supporting the utility of this type of sample for diagnostic and epidemiological studies. Furthermore, oral fluid collection is non-invasive and therefore advantageous over serum as a tool for detection and monitoring of roseolovirus infection [3,14,21].

Persistence of Roseoloviruses in the salivary glands has been reported [12]. However, earlier studies did not distinguish between the prevalence of infection (viral load) and active replication (mRNA detection), here we evaluated the presence of viral load and active infection in three moments, before and after kidney transplantation, the detection of HHV-6 mRNA ranged from 3.1% to 18.8%. In our results, HHV-6 showed an increase in replication in T2 and T3, although without statistical significance. A previous study that investigated the presence of active infection samples of Peripheral Blood Mononuclear Cell (PBMC) and thyroid gland in 45 autoimmune thyroiditis patients, using the same target region to mRNA that we used, found that 41% samples from thyroid glands were positive for HHV-6 mRNA, and no replication was detected PBMC [19]. Another study with 27 patients detected the presence of mRNA in PBMC samples and found 3.7–33% positivity [22]. These results showed that the HHV-6 mRNA rate can be lower than the detection of HHV-6 DNA; and suggested that HHV-6 replication can happen in PBMC, salivary glands and thyroid glands, however, more studies should be done to investigated which is the main HHV-6 replication site.

HHV-7 showed a significant increase in viral replication at T3 compared to T1, although there are no studies of detection of mRNA for HHV-7, its replication is very similar to that of HHV-6 and the coinfection of Roseoloviruses has already been described in immunosuppressed patient cases [23]. HHV-6 and HHV-7 are characterized by widespread tropism in various types of human cells. Therefore, the need to research different replication and persistence sites is important. In addition, comparing our findings with other studies, it is clear that there is a difference in the prevalence of active infection in each sample surveyed and type of virus.

Analysis of the ratio between viral and quantitative replicants (DNA copies/mL) showed that positive cases for viral replication had a higher number of DNA copies (>10^6^) when compared to cases without replication for both HHV-6 and HHV-7 (p < 0.001), being a statistically significant result for HHV-7. Interestingly, high viral loads, assessed based on DNA levels, were correlated with mRNA detection levels, suggestive of active replication of HHV-6 and HHV-7. A previous study reported high levels of HHV-6 DNA in body fluids of individuals with persistent infection (usually millions of genomic copies) relative to only tens of thousands of viral copies in patients with acute HHV-6 infection or reactivation, even in immunocompromised cases [24].

These data are important for monitoring active infection in renal transplant patients. Our results suggest that HHV-6 and HHV-7 replication occurs in salivary glands and although the replication processes of both viruses are independent, there is a positive synergy between them. Comparison of viral loads revealed a simultaneous increase in replication.

Ganciclovir and valganciclovir are antiviral drugs commonly used to treat betaherpesviruses [25]. While we observed no significant association between treatment with these drugs and viral load, decreased HHV-6 and HHV-7 viral loads were observed in patients administered the drugs.

Previous studies have shown variable viral DNA detection across different geographic areas and socioeconomic groups [3,14,26]. In solid organ recipients, reactivation of roseoloviruses is frequent and usually asymptomatic although clinical complications may arise. Both HHV-6 and 7 are highly prevalent in the healthy population, establish latency in macrophages and T-lymphocytes, and are intermittently shed in the saliva of healthy donors. The pathogenic potential of reactivated virus ranges from asymptomatic infection to severe disease in transplant recipients [27]. Primary infections during the first year after transplantation have been reported in adult solid organ transplant patients. In addition, roseoloviruses have been detected in post-transplant patients with chronic kidney disease [28].

Detection of HHV-6 and HHV-7 mRNA in both pre- and post-renal transplant patients highlights the salivary gland as an important site of viral infection, especially in immunosuppressed patients. This is the first report of the detection of HHV-6 and HHV-7 mRNA in oral fluid after renal transplantation, suggestive of active replication. Evidence of roseolovirus mRNA or high viral load over time is clearly indicative of replication with important implications for the diagnosis and monitoring of high-risk patients.
